# Extending the tempotron with hierarchical dendrites allows faster learning 

**DOI:** 10.1186/1471-2202-16-S1-P37

**Published:** 2015-12-18

**Authors:** Sarah J Jarvis, Romain Caze, Claudia Clopath

**Affiliations:** 1 Department of Bioengineering, Imperial College London, SW7 2AZ, UK

## 

Certain functional classes of neurons seem to be able to differentiate between input patterns with high temporal precision. As input patterns to neurons can consist of up to thousands of inputs, the ability to identify target patterns amongst statistically similar background patterns is impressive and has been suggested to occur via modification to synaptic weights. Yet how does learning that occurs locally at the level of synapses converge, without global coordination? While this question is important for understanding how synaptic learning gives rise to dendritic computation, it is impractical to test experimentally. Insight from abstract neuronal models, such as the multilayer perceptron networks [1], provide a potential glimpse of the difficulty of how to ensure that global convergence during learning when weight changes are local. In this work, we report that by extending the tempotron model [2], we are able to demonstrate that learning can, indeed, learn locally but also converge globally. By arranging dendritic units in a hierarchical manner which feeds into a master dendrite and soma, learning occurs over two timescales: locally on each dendritic branch, using a simple incremental plasticity rule; and at a slower timescale on the main branch, where information is integrated across branches. We observe that the inclusion of dendrites reduces the learning time required by allowing dendrites to subsample the entire input space. In comparison to one single dendrite receiving *n *inputs, the inclusion of *m *dendrites means that each dendrite is now subsampling *n/m *inputs, which not results in faster learning epochs before convergence but also improves the overall robustness against noise. Thus, the move from a single tempotron to a set of hierarchically configured tempotrons, representing dendrites, imbues the unit with recognition of pattern fragments (with a pattern capacity > *m*!*)*, faster convergence during learning and increased noise tolerance (both of which scale with *m*). The inclusion of dendrites also allows for them to signal in sequences with varying relative temporal offsets, granting the neuron the opportunity to differentiate between multiple positive patterns to identify not only *which *pattern was observed but also *when*. Furthermore, we have also demonstrated that tempotrons can be extended to work for non-episodic patterns i.e. ongoing and without reset, and can also perform well when number of distractor patterns greatly outnumber positive patterns. Conceptually, our model reconciles the tempotron learning rule with work on dendritic computation which argues for dendrites as computation units [3]. It also provides a potential explanation to phenomena observed experimentally, such as neurons in visual cortex whose dendrites had other preferred orientations that were different to those of the soma [4].
